# Remimazolam for Procedural Sedation in Older Patients: A Systematic Review and Meta-Analysis with Trial Sequential Analysis

**DOI:** 10.3390/jpm14030276

**Published:** 2024-02-29

**Authors:** Myeongjong Lee, Cheol Lee, Guen Joo Choi, Hyun Kang

**Affiliations:** 1 Department of Anesthesiology and Pain Medicine, Research Institute of Medical Science, Konkuk University Medical School, 82 Guwondae-ro, Chungju 27376, Republic of Korea; gooddr21@kku.ac.kr; 2 Department of Anesthesiology and Pain Medicine, Wonkwang University School of Medicine, 895 Muwang-ro, Iksan 54538, Republic of Korea; ironyii@wku.ac.kr; 3 Department of Anesthesiology and Pain Medicine, Chung-Ang University College of Medicine, 84 Heukseok-ro, Dongjak-gu, Seoul 06911, Republic of Korea; pistis23@naver.com

**Keywords:** aged, sedation, endoscopy, remimazolam, sedatives, systematic review

## Abstract

This systematic review and meta-analysis with trial sequential analysis (TSA) aimed to evaluate the efficacy and safety of remimazolam compared to other sedatives for procedural sedation in older patients. We registered the protocol of this systematic review and meta-analysis with TSA in the PROSPERO network (CRD42023441209). Two investigators performed a systematic, comprehensive, and independent search of the PubMed, EMBASE, and Cochrane Central Register of Controlled Trials databases to identify randomized controlled trials (RCTs) comparing remimazolam with other sedatives in older patients undergoing procedural sedation. Conventional meta-analysis and TSA were also performed. Seven RCTs (1502 patients) were included. Pooled results demonstrated that remimazolam was associated with a low incidence of hypoxemia, hypotension, bradycardia, respiratory depression, and injection pain. Remimazolam also required a long time to cause loss of consciousness. There were no differences in rates of sedation success, dizziness/headache, postoperative nausea and vomiting, or recovery time. Older patients receiving procedural sedation with remimazolam had a lower risk of hypoxemia, hypotension, bradycardia, respiratory depression, and injection pain than those receiving other sedatives, suggesting that remimazolam may be more suitable for procedural sedation in older patients.

## 1. Introduction

Recently, there has been a consistent rise in endoscopic procedures driven by advancements in medical technology, an increased demand for preventive healthcare, and a growing aging population [1,2,3]. Procedural sedation is widely used during endoscopy to reduce patients’ anxiety, discomfort, and pain, and propofol, etomidate, and midazolam are the most commonly used agents [4,5]. However, these drugs have disadvantages, such as cardiovascular and respiratory depression, injection pain, muscle tremor and rigidity, lack of specific antagonists, and interindividual variability [6,7,8]. 

With the aging population, the number of procedures performed on older people is also increasing. Furthermore, because older people are more susceptible to the side effects of sedative medication, including morbidity and mortality, clinicians should pay special attention to circulatory and respiratory depression during procedures performed on older people. The ideal agent for procedural sedation in older patients needs to be safe and effective throughout the process. Remimazolam, a novel ultra-short-acting benzodiazepine, is characterized by mild respiratory suppression, hemodynamic stability, and rapid onset and offset [9]. While most studies have focused on adults in general, limited research has explored the effects of remimazolam in older patients [10,11,12,13,14,15,16]. Furthermore, meta-analyses comparing the efficacy and safety of remimazolam with other sedatives during procedural sedation in older patients are lacking. 

Consequently, we conducted a systematic review and meta-analysis with a trial sequential analysis (TSA) of randomized controlled trials (RCTs) to clarify the efficacy and safety of remimazolam compared to other sedatives for procedural sedation in the older population.

## 2. Materials and Methods

We developed a protocol for this systematic review and meta-analysis with TSA according to the Preferred Reporting Items for Systematic Review and Meta-Analysis Protocol (PRISMA-P), and registered it in the PROSPERO network (registration number: CRD42023441209; https://www.crd.york.ac.uk/PROSPERO/display_record.php?RecordID=441209 accessed on 29 February 2024) on 11 July 2023. This study was conducted based on the recommendations of the Cochrane Collaboration [17,18] and was reported following the PRISMA statement guidelines [19].

### 2.1. Inclusion and Exclusion Criteria

The inclusion and exclusion criteria were determined before conducting the systematic search. We included full reports of RCTs that compared remimazolam with other sedatives for procedural sedation in older patients.

The participants, intervention, comparison, outcome, and study design (PICO-SD) information was as follows:

(1) Patients (P): all older patients undergoing elective procedural sedation
(2) Intervention (I): remimazolam bolus or continuous infusion
(3) Comparison (C): other sedatives
(4) Outcome measurements (O): The primary outcome of this systematic review and meta-analysis with a TSA was the rate of sedation success, which was defined as no rescue sedation with a sedative agent other than the assigned treatment to complete the entire endoscopic procedure. The incidence of hypoxemia (SpO_2_ < 90%) and hypotension (systolic blood pressure [SBP]) decreased by 20% or more compared to the baseline value or SBP ≤ 80 mmHg during the procedure. The secondary outcomes were the rates of bradycardia (≤50 beats/min or a decrease in heart rate of 20% or more from baseline), respiratory depression (respiratory rate < 8/min and/or SpO_2_ < 90%), time to loss of consciousness (LOC), recovery time (time from cessation of sedative agent to fully alert), injection pain, dizziness/headache, and postoperative nausea and vomiting (PONV).
(5) Study design (SD): Full reports of RCTs were included. Observational studies, conference abstracts, posters, case reports, case series, comments, letters to the editor, reviews, and laboratory or animal studies were excluded.

### 2.2. Information Source and Search Strategy

To identify RCTs for this systematic review and meta-analysis with a TSA, two investigators (ML and GC) independently searched the PubMed, EMBASE, and Cochrane Central Register of Controlled Trials (CENTRAL) databases on 28 July 2023. The search terms included the following in various combinations with free text, Medical Subject Headings, and EMTREE terms including “old”, “aged”, “elderly”, “remimazolam”, “endoscopy”, “procedure”, “sedation”, “outpatient”, “bronchoscopy”, and “colonoscopy”. No limitations were placed on publication date or language.

### 2.3. Study Selection

Two investigators (ML and CL) independently scanned the titles and abstracts of the reports identified. If a report was considered eligible based on the title or abstract, the full text was retrieved and evaluated. All abstracts that did not provide sufficient information regarding the eligibility criteria were selected for full-text evaluation. Potentially relevant studies identified by at least one investigator were retrieved and the full-text versions were evaluated. Full-text versions that met the inclusion criteria were assessed separately by two investigators (ML and CL), and any discrepancies were resolved by discussion. Disagreements regarding inclusion or exclusion were resolved by discussion with a third investigator (HK).

Kappa statistics were used to measure the degree of agreement for study selection between the two independent investigators. Kappa statistics were interpreted as follows: (1) less than 0, less than chance agreement; (2) 0.01–0.20, slight agreement; (3) 0.21–0.40, fair agreement; (4) 0.41–0.60, moderate agreement; (5) 0.61–0.80, substantial agreement; and (6) 0.8–0.99, almost perfect agreement [20].

### 2.4. Data Extraction

Using a standardized data collection form, two independent investigators (ML and GC) extracted all relevant data from the included studies, entered them into standardized forms, and then crosschecked them. Any discrepancy was resolved through discussion. If agreement could not be reached, a third investigator (HK) provided a resolution. The extracted data included the first author, journal, publication year, country of origin, study protocol registration (registry and registration number), study design, sedation success rate, incidence of hypoxemia and hypotension, bradycardia, respiratory depression, time to LOC, recovery time, injection pain, dizziness, headache, and PONV. The data were initially extracted from tables or text. In cases of missing or incomplete data, the authors were contacted to obtain the relevant information.

### 2.5. Risk of Bias

The risk of bias was assessed by two independent authors (ML and HK) using the Revised Cochrane risk of bias tool for RCTs (RoB 2.0) version (22 August 2019). RoB 2.0 was structured into five domains: D1, bias arising from the randomization process; D2, bias due to deviations from the intended interventions; D3, bias due to missing outcome data; D4, discrimination in the measurement of the outcome; and D5, bias in the selection of reported results. The overall risk of bias was assessed. Risk was judged as low when the risk of bias for all domains was low, high when the risk of bias for at least one domain was high or when the risk of bias for multiple domains was of some concern, and some concern if the overall judgment was neither low nor high.

### 2.6. Data Analysis

#### 2.6.1. Conventional Meta-Analysis

A meta-analysis was conducted using Comprehensive Meta-Analysis (version 2.0; Englewood, NJ, USA, 2008). Two investigators (ML and HK) independently entered all data into the software. The pooled risk ratio (RR) for binary variables, the weighted mean difference (WMD) for quantitative variables, and their 95% confidence intervals (CIs) were calculated for each outcome. Heterogeneity between studies was evaluated using the I^2^, *τ*, and 95% predictive interval (PI). If τ was 0.00, PI was not calculated. If heterogeneity was suspected, a random-effects model was used; otherwise, a fixed-effects model was used. We performed a sensitivity analysis to explore the heterogeneity by removing one study at a time and evaluating whether it altered our results. Publication bias was not estimated because fewer than 10 studies were included. To estimate the overall clinical impact of the intervention, we calculated the number needed to treat (NNT) using a 95% CI based on the absolute risk reduction.

#### 2.6.2. TSA

Conventional meta-analyses run the risk of random errors owing to sparse data. TSA is a methodology that includes the required information size (RIS) calculation for meta-analyses with a threshold for statistical significance, which controls the risk of potential false-positive and false-negative findings in meta-analyses [21]. Therefore, we performed TSA on the outcomes to calculate the RIS and assess whether our results were conclusive. We used a random-effects model with the DerSimonian–Laird (DL) method to construct a cumulative Z-curve. TSA was performed to maintain an overall 5% risk of type I errors. When the cumulative Z-curve crossed the trial sequential monitoring boundary or entered the futility area, a sufficient level of evidence for accepting or rejecting the anticipated intervention effect was reached and no further studies were needed. If the Z-curve did not cross any boundaries and the RIS was not reached, the evidence to reach a conclusion was insufficient, indicating the need for further studies. For dichotomous outcomes, we estimated the RIS based on the observed proportion of patients with an outcome in the remimazolam group (the cumulative proportion of patients with an event relative to all patients in the other sedative groups), a relative risk reduction of 30% in the remimazolam group, an alpha of 5% for all outcomes, a beta of 20%, and observed diversity, as suggested by the trials in the meta-analysis.

For quantitative outcomes, we used the observed standard deviation (SD) in the TSA, the mean difference of the observed SD/3, an alpha of 5% for all outcomes, a beta of 20%, and the observed diversity, as suggested by the trials in the meta-analysis.

#### 2.6.3. Quality of the Evidence

The evidence grade was determined using the Grading of Recommendations Assessment, Development, and Evaluation (GRADE) system, which uses a sequential assessment of evidence quality, followed by an assessment of the risk–benefit balance and a subsequent judgment on the strength of the recommendations [22].

## 3. Results

### 3.1. Study Selection

**Figure 1:**
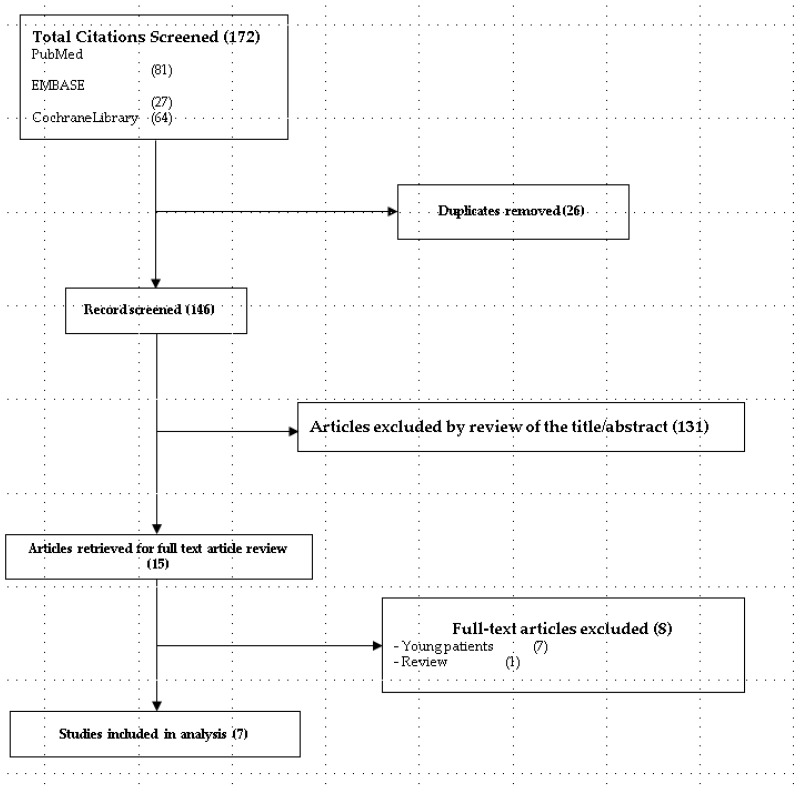
Flow diagram showing the number of abstracts and articles identified and evaluated during the review process.

From the PubMed, EMBASE, and CENTRAL databases, 172 studies were selected. After adjusting for duplicates (*n* = 26), 146 studies were included. Of these, 131 were excluded after reviewing their titles and abstracts because they were not relevant. At this stage, the kappa value for the selection of studies by the two reviewers was 0.759. Full texts of the remaining 15 studies were reviewed in detail. Of these, eight studies were further excluded because seven studies did not include older patients [23,24,25,26,27,28,29] and one study was a review [30]. The kappa value for the articles selected by the two investigators was 0.875. Finally, seven studies with 1502 patients satisfied the inclusion criteria and were included in our meta-analysis (Figure 1). One study did not report separate events, such as hypotension and bradycardia, but rather combined hemodynamic events [12]. Thus, we attempted to contact the authors for accurate information; however, we could not obtain accurate information.

### 3.2. Study Characteristics

Study characteristics are presented in Table 1. Only one study used etomidate plus propofol (10 mL of 20 mg etomidate plus 10 mL of 100 mg propofol) [10], whereas others used propofol as a control drug [11,12,13,14,15,16]. All patients underwent gastroscopy, colonoscopy, or both. The primary outcomes of the four studies were hypoxemia and/or hypotension [13,14,15,16], recovery of the cognitive domain [11], and success of the procedure [10,12]. Two studies were conducted in patients with American Society of Anesthesiology (ASA) class I–III [13,14], and five studies were conducted in patients with ASA I–II [10,11,12,15,16].

All the studies were conducted in China and registered in the Chinese Clinical Trial Registry. 

### 3.3. Risk of Bias

The risk of bias assessment, performed using the Cochrane tool for the included studies, is shown in Table 2. Most studies were assessed as “low risk” in all domains, except for bias arising from the randomization process in which four studies [10,11,15,16] were assessed as “some concern.” Overall bias was assessed as “low risk” in three studies [12,13,14] and “some concern” in four studies [10,11,15,16].

### 3.4. Sedation Success Rate 

All studies [10,11,12,13,14,15,16] (1502 patients) measured the sedation success rates. There was no evidence of a difference in the sedation success rate between remimazolam and propofol groups (RR: 0.999; 95% CI 0.995–1.003; I^2^ = 0.0; τ = 0.000, NNTH: 96; 95% CI NNTH 56 to ∞ NNTB 351) (Figure S1).

The TSA indicated that the number of accrued patients exceeded the RIS (1502 of 599 patients) (Figure S2). 

### 3.5. Incidence of Hypoxemia

**Figure 2:**
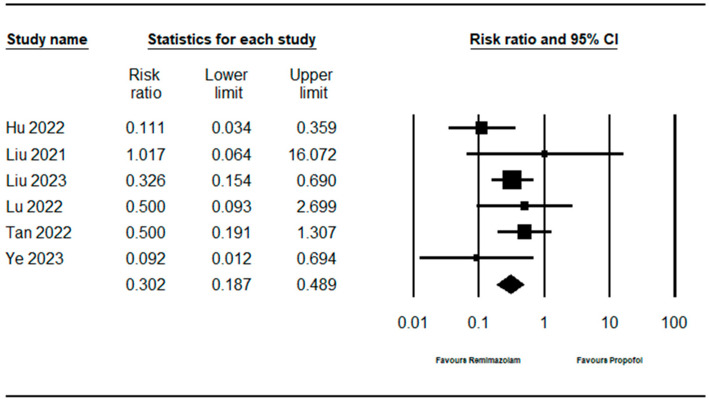
Forest plot for the studies comparing the effect of remimazolam to that of propofol on the incidence of hypoxemia. The figure depicts individual trials as filled squares with relative sample size and the 95% confidence interval (CI) of the difference as a solid line. The diamond shape indicates the pooled estimate and uncertainty for the combined effect [10,11,13,14,15,16].

Six studies [10,11,13,14,15,16] (1422 patients) measured the incidence of hypoxemia. The incidence of hypoxemia in the remimazolam group was significantly lower than that in the propofol group (RR: 0.302; 95% CI 0.187–0.489; I^2^ = 20.595; τ = 0.573, 95% PI 0.069–1.316, NNTB: 13; 95% CI NNTB 10 to NNTB 20) (Figure 2, Table 3).

**Figure 3:**
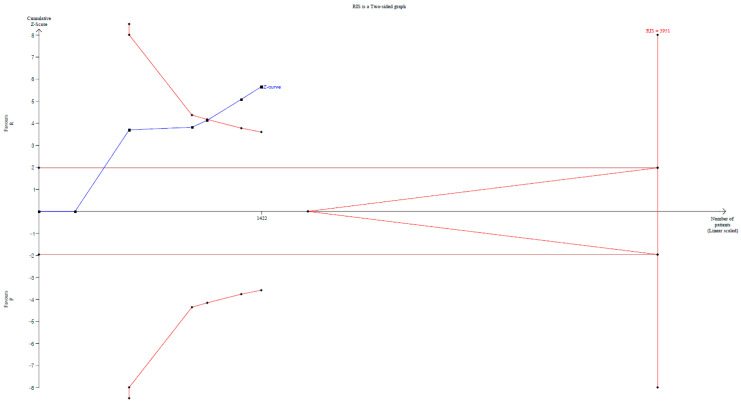
Trial sequential analysis for the studies comparing the effect of remimazolam to that of propofol on the incidence of hypoxemia. Uppermost and lowermost curves represent trial sequential monitoring boundary lines for benefit and harm, respectively. Horizontal line represents the conventional boundaries for statistical significance. Triangular lines on the right side reflect the futility boundaries. The blue solid line represents the cumulative Z-curve. The number on the *x*-axis indicates required information size.

The TSA indicated that only 36.0% (1422 of 3951 patients) of the RIS was met. The cumulative Z-curve crossed both the conventional test boundary and the trial sequential monitoring boundary (Figure 3).

### 3.6. Incidence of Hypotension

**Figure 4:**
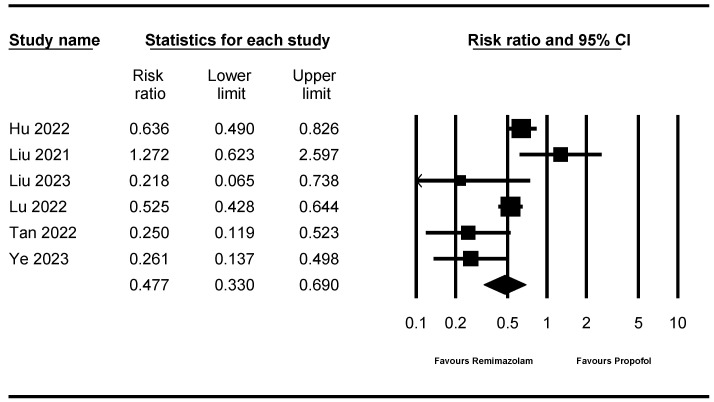
Forest plot for the studies comparing the effect of remimazolam to that of propofol on the incidence of hypotension. The figure depicts individual trials as filled squares with relative sample size and the 95% confidence interval (CI) of the difference as a solid line. The diamond shape indicates the pooled estimate and uncertainty for the combined effect [10,11,13,14,15,16].

Six studies [10,11,13,14,15,16] (1422 patients) measured the incidence of hypotension. The incidence of hypotension in the remimazolam group was significantly lower than that in the propofol group (RR: 0.477; 95% CI 0.330–0.690; I^2^ = 72.634, τ = 0.593, 95% PI 0.104–2.192; NNTB: 5; 95% CI NNTB 4 to NNTB 6) (Figure 4). Sensitivity analysis, performed by removing one study at a time, showed no statistically significant changes (Figure S3).

**Figure 5:**
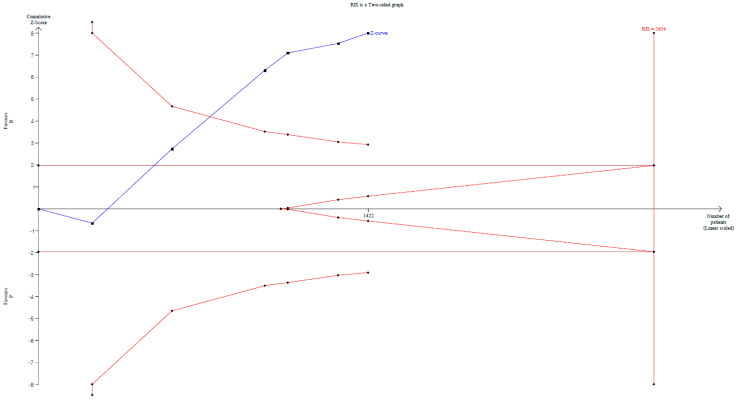
Trial sequential analysis for the studies comparing the effect of remimazolam to that of propofol on the incidence of hypotension. Uppermost and lowermost curves represent trial sequential monitoring boundary lines for benefit and harm, respectively. Horizontal line represents the conventional boundaries for statistical significance. Triangular lines on the right side reflect the futility boundaries. The blue solid line represents the cumulative Z-curve. The number on the *x*-axis indicates required information size.

The TSA indicated that only 53.6% (1422 of 2654 patients) of the RIS was accrued. The cumulative Z-curve crossed both the conventional test boundary and the trial sequential monitoring boundary (Figure 5).

### 3.7. Incidence of Bradycardia

Five studies [10,13,14,15,16] (1323 patients) measured the incidence of bradycardia. The incidence of bradycardia in the remimazolam group was significantly lower than that in the propofol group (RR: 0.396; 95% CI 0.249–0.631; I^2^ = 47.484; τ = 0.722, 95% PI 0.053–2.944, NNTB: 16; 95% CI NNTB 11 to NNTB 29) (Figure S4).

The TSA indicated that only 22.6% (1323 of 5851 patients) of the RIS was accrued. 

The cumulative Z-curve crossed the conventional test boundary but not the trial sequential monitoring boundary (Figure S5).

### 3.8. Respiratory Depression

Four studies [10,12,13,16] (838 patients) measured the incidence of respiratory depression. The incidence of respiratory depression in the remimazolam group was significantly lower than that in the propofol group (RR: 0.347; 95% CI 0.183–0.659; I^2^ = 0.0; τ = 0.000, NNTB: 17; 95% CI NNTB 11 to NNTB 36) (Figure S6).

The TSA indicated that only 26.9% (838 of 3118 patients) of the RIS was accrued. 

The cumulative Z-curve crossed the conventional test boundary but not the trial sequential monitoring boundary (Figure S7).

### 3.9. Time to LOC

Four studies [12,13,14,16] (952 patients) measured the time to LOC. The time to LOC in the remimazolam group was significantly longer than that in the propofol group (WMD: 5.385; 95% CI 2.082–8.689; I^2^ = 65.420, τ = 1.641, 95% PI 0.163–10.607) (Figure S8). Sensitivity analysis, performed by removing the study by Guo et al., changed the statistical significance of the results without eliminating heterogeneity (I^2^ = 65.408) (Figure S9).

The TSA indicated that the number of patients almost reached the RIS (952 of 957 patients). 

The cumulative Z-curve crossed both the conventional test and trial sequential monitoring boundaries (Figure S10).

### 3.10. Recovery Time

**Figure 6:**
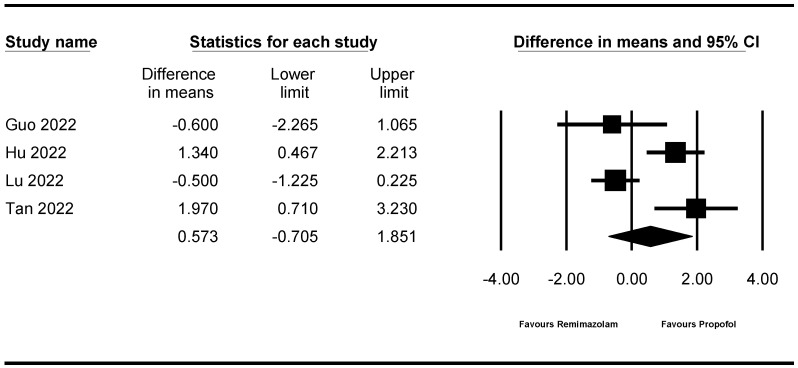
Forest plot showing recovery time. The figure depicts individual trials as filled squares with relative sample size and the 95% confidence interval (CI) of the difference as a solid line. The diamond shape indicates the pooled estimate and uncertainty for the combined effect [11,12,13,14].

Four studies [11,12,13,14] (922 patients) measured recovery time. There was no evidence of a difference in the recovery time between the remimazolam and propofol groups (WMD: 0.573; 95% CI −0.705–1.851; I^2^ = 83.050, τ = 1.080, 95% PI −2.865–4.011) (Figure 6). Sensitivity analysis, performed by removing one study at a time, showed no statistically significant changes (Figure S11).

The TSA indicated that only 46.8% (922 of 1968 patients) of the RIS was accrued. 

The cumulative Z-curve did not cross the conventional test boundary (Figure S12). 

### 3.11. Injection Pain

Four studies [10,12,13,16] (838 patients) measured the incidence of injection pain. The incidence of injection pain in the remimazolam group was significantly lower than that in the propofol group (RR: 0.207; 95% CI 0.074–0.579; I^2^ = 2.823, τ = 0.628, 95% PI 0.028–1.526) (Figure S13). However, the results from the NNT did not show statistical significance (NNTB: 428; 95% CI NNTH 86 to ∞ NNTB 61).

The TSA indicated that only 3.8% (838 of 22,319 patients) of the RIS was accrued. 

The cumulative Z-curve crossed the conventional test boundary but did not cross the trial sequential monitoring boundary (Figure S14).

### 3.12. Incidence of Dizziness/Headache

Four studies [12,13,15,16] (822 patients) measured the incidence of dizziness/headache. There was no evidence of a difference in the incidence of dizziness/headache between the remimazolam and propofol groups (RR: 0.726; 95% CI 0.419–1.257; I^2^ = 0.0; τ = 0.000, NNTB: 60; 95% CI NNTH 77 to ∞ NNTB 21) (Figure S15).

The TSA indicated that only 17.2% (822 of 4774 patients) of the RIS was accrued. 

The cumulative Z-curve did not cross the conventional test boundary (Figure S16).

### 3.13. PONV

Four studies [12,13,14,15] (1039 patients) measured the incidence of PONV. There was no evidence of a difference in the PONV between the remimazolam and propofol groups (RR: 1.411; 95% CI 0.778–2.559; I^2^ = 0.0; τ = 0.000, NNTH: 74; 95% CI NNTH 28 to ∞ NNTB 111) (Figure S17).

The TSA indicated that only 10.4% (1039 of 9973 patients) of the RIS was accrued. 

The cumulative Z-curve did not cross the conventional test boundary (Figure S18).

### 3.14. Quality of Evidence

Ten outcomes were evaluated using the GRADE system (Table 4). The quality of the pooled analysis for sedation success rate, hypoxemia, and bradycardia was graded as high. The quality of the pooled analysis of the time to LOC and recovery times was low. Otherwise, the quality of the pooled analyses was graded as moderate.

## 4. Discussion

This meta-analysis of seven RCTs, including 1502 patients (of whom 767 received remimazolam, 618 received propofol, and 117 received etomidate plus propofol), demonstrated that remimazolam had lower incidences of hypoxemia and hypotension and a longer time to LOC than propofol. In these outcomes, the cumulative Z-curve crossed the trial sequential monitoring boundary, suggesting that the TSA results reached a sufficient level of evidence and were conclusive.

This meta-analysis also showed that remimazolam was associated with a lower incidence of bradycardia, respiratory depression, and injection pain than propofol. However, the cumulative Z-curve did not cross the trial sequential monitoring boundary because of the sparse data. Regarding the incidence of dizziness/headache, PONV, sedation success, and recovery time, there was no evidence of differences between the conventional meta-analysis and TSA. 

Remimazolam, which acts on gamma-aminobutyric acid receptors, is a novel, ultrashort-acting benzodiazepine that may provide a new direction for procedural sedation. It contains a carboxyl ester bond and can be rapidly hydrolyzed into an inactive metabolite by human tissue esterases [9]. 

Several RCTs have compared remimazolam with other sedatives used for procedural sedation in older patients [10,11,12,13,14,15,16]; however, to date, their findings have not been pooled. Therefore, we conducted this study to provide necessary evidence to guide the administration of sedatives in the older population. The increase in the geriatric population has brought about profound changes to the healthcare landscape. Diagnostic and therapeutic procedures are becoming increasingly minimally invasive and their scope is expanding with advances in medical technology. The expansion of the scope has also led to an increase in the need for procedural sedation and analgesia. This change in the healthcare environment is favorable for older patients who are likely to have a relatively large number of comorbidities and are at a high risk of respiratory and cardiovascular complications. However, for healthcare providers providing sedation during procedures, increasing the age and severity of underlying diseases in older patients remains very challenging. 

Hypoxemia and hypotension during procedural sedation are not unusual adverse events [31]. Older patients are more sensitive to sedatives because of their slower metabolism, lower physiological reserve, and smaller volume of distribution [32]. Therefore, clinicians should pay close attention to hemodynamic events and respiratory depression during procedural sedation. In our study, remimazolam significantly decreased the incidence of hypoxemia and hypotension compared to propofol in both the conventional meta-analysis and TSA. Furthermore, a conventional meta-analysis has shown that remimazolam decreases the incidence of bradycardia and respiratory depression. These results support that remimazolam is more advantageous than propofol from a drug safety perspective in older patients.

The issue of safety is of paramount importance when performing procedural sedation in older patients because even for the same medical condition, the margin of safety narrows with advanced age. Given this, the finding of the superior safety of remimazolam compared to propofol, the most widely used sedative, in this study is clinically significant. This evidence provides clinicians with a favorable option for sedation selection when providing procedural sedation to older patients in clinical practice. However, care should be exercised when extrapolating and applying these findings to clinical settings. While this systematic review and meta-analysis with trial sequential analysis encompassed numerous studies employing a high initial bolus of propofol, real-world clinical situations, especially involving older patients, often warrant a smaller initial bolus dose of propofol, with subsequent dosing adjustments based on observed clinical effects. Such an approach aids in reducing propofol-related adverse events like hypotension, bradycardia, respiratory depression, and hypoxemia. The utilization of a high initial dose of propofol in the propofol group may potentially introduce bias favoring the remimazolam group.

In terms of sedation efficacy, there were no differences in the sedation success rate and recovery time. Our study revealed that the time to LOC was slightly longer with remimazolam than with propofol (WMD, 5.385; 95% CI, 2.082–8.689). However, this difference was not clinically significant. Although propofol has some safety concerns, it is known to be an excellent sedative with respect to its effectiveness, as it has a rapid onset and offset and facilitates the induction of sedation at an appropriate depth, which can increase the success rate of procedures and sedation. The finding in this study that remimazolam was noninferior to propofol in procedural sedation of older patients in terms of procedural success rate and recovery time is noteworthy. Delayed recovery time can be a concern, especially in older patients. The evidence from this study that remimazolam is comparable in efficiency to propofol should be a decisive advantage in the use of procedural sedation.

There appears to be a lack of evidence regarding remimazolam and delirium in older patients. Further evidence related to delirium in older patients and procedural sedation, such as general anesthesia in clinical practice, is essential for clarification of the pros and cons of using remimazolam for procedural sedation in older patients. Yang et al. reported that general anesthesia with remimazine in older patients undergoing orthopedic surgery did not result in an increase in postoperative delirium compared with propofol [33]. Recently, research on remimazolam has been increasing. Remimazolam’s advantages, such as fast onset and recovery, mild respiratory suppression, hemodynamic stability, and independent metabolism of the liver and kidney, enable better sedation than others for procedural sedation not only in older patients but also in critical care patients with hepatic or renal impairment [34]. Oyosi et al. and Kido et al. also reported favorable outcomes using remimazolam for general anesthetic management in critically ill patients [35,36]. However, owing to the limited number of studies, further trials are needed to determine whether remimazolam is safe for anesthesia in critically ill patients.

Our study had some limitations. First, although we aimed to compare the outcomes of remimazolam and other sedatives in older patients undergoing all types of procedures, only gastroscopy and colonoscopy procedures using propofol (one patient was treated with etomidate plus propofol) were accessible. Second, all the included studies were conducted in China. To generalize the results of our study, more research involving patients of other ethnicities should be conducted. However, the characteristics of the literature that were finally included studies made the data in this study less heterogeneous. Heterogeneity is a fundamental limitation of meta-analyses, and we believe that the types of procedures and ethnicities of participants in each study were less heterogeneous than those in other meta-analyses, which may increase the reliability of our findings. The comparator sedative was propofol in all included studies, of which one study used a combination of propofol and etomidate. However, a sensitivity analysis excluding this study did not change the significance of the results. Lastly, most studies included relatively healthy older patients and excluded those with significant cardiopulmonary instability, clinically significant hepatic or renal impairment, history of sleep apnea, body mass index >30 kg/m^2^, or expected difficult airways. Only two studies included in our systematic review and meta-analysis with trial sequential analysis encompassed ASA class III [13,14]. Therefore, applying this method to critically ill older patients may be challenging. There are inherent risks associated with advanced age and this factor should be weighed against risks and benefits when making medical decisions. This study provides evidence to guide the use of sedatives during procedural sedation in vulnerable older patients. TSA was applied to determine whether the current results were conclusive and whether further research is needed, thereby providing directions for future research. 

## 5. Conclusions

Older patients receiving procedural sedation with remimazolam had a lower risk of hypoxemia, hypotension, bradycardia, respiratory depression, and injection pain than those receiving other sedatives, suggesting that remimazolam may be more suitable for procedural sedation in older patients. However, the safety of remimazolam remains uncertain due to limitations within the studies included. Further investigation, specifically targeting sick older patients (such as those classified as ASA III or IV) and employing smaller doses of propofol, is essential to definitely establish its safety profile.

## Figures and Tables

**Table 1:** Characteristics of included studies.

Author, Year, Country	Participants			Sample Size/Intervention	Primary Outcome	Major Finding
	Age (Year)	Procedure	ASA			
Liu et al., 2021, China [10]	65–75	Colonoscopy	I–II	Remimazolam (*n* = 115) initial bolus 0.15 mg/kg (over 1 min), followed by 0.075 mg/kg Propofol plus etomidate (*n* = 117) bolus 0.1 mL/kg (over 1 min), followed by 0.05 mL/kg	Success of the procedure	Remimazolam may have noninferior efficacy and a higher safety profile than etomidate-propofol in older colonoscopy
Tan et al., 2022, China [11]	>60	Upper GI endoscopy	I–II	Remimazolam (*n* = 66) initial bolus 0.1 or 0.2 mg, followed by 0.05 mg/kg Propofol (*n* = 33) bolus 1.0–1.5 mg/kg, followed by 0.5 mg/kg	Recovery of the cognitive domain	Remimazolam is a safe and effective sedative in older upper GI endoscopy
Lu et al., 2022, China [13]	65–85	Upper GI endoscopy	I–III	Remimazolam (*n* = 200) 300 mg/h, followed by 2.5 mg Propofol (*n* = 200) 3.0 g/h, followed by 0.5 mg/kg	Rate of hypotension, defined as SBP ≤ 90 mmHg or a >30% decline from the baseline	Remimazolam is associated with a low rate of hypotension in older upper GI endoscopy
Hu et al., 2022, China [14]	≥65	Gastroscopy	I–III	Remimazolam (*n* = 173) initial bolus 0.2 mg/kg (slow IV injection, over 1 min), followed by one-third of the initial dose Propofol (*n* = 173) initial bolus 1.5 mg/kg (slow IV injection, over 1 min), followed by one-third of the initial dose	Incidence of respiratory depression, defined as respiratory rate < 8/min and/or oxygen saturation <90%	The incidence of respiratory depression is significantly reduced in patients administered remimazolam compared with the patients administered propofol
Guo et al., 2022, China [12]	≥65	GI endoscopy	I–II	Remimazolam (*n* = 39) initial bolus 0.15 mg/kg (slow IV injection, completed in 30 s), followed by one-third of the initial dose Propofol (*n* = 38) initial bolus 1.5 mg/kg (slow IV injection, completed in 30 s), followed by one-third of the initial dose	Success rate of sedation, the time to loss of consciousness, the recovery time between the remimazolam and propofol	Compared with propofol, remimazolam can be safely and effectively used in older GI endoscopy
Ye et al., 2023, China [16]	≥65	Gastroscopy	I–II	Remimazolam (*n* = 64) initial bolus 0.2 mg/kg, followed by 3 mg Propofol (*n* = 65) initial bolus 2.0 mg/kg, followed by 30 mg	Incidence of adverse events to assess safety (hypotension, hypertension, bradycardia, tachycardia, respiratory depression, hypoxia, injection pain)	Remimazolam is safer alternative than propofol in older gastroscopy
Liu et al., 2023, China [15]	60–80	GI endoscopy	I–II	Remimazolam (*n* = 107) initial bolus 0.10 mg/kg, followed by 2.5 mg Propofol (*n* = 109) initial bolus 1.5 mg/kg, followed by 0.5 mg/kg	Incidence of moderate hypoxemia, defined as SpO_2_ ≥ 85% and <90%, duration > 15 s	Remimazolam improves risk of moderate hypoxemia and hypotension in older GI endoscopy

ASA, American Society of Anesthesiology classification; *n*, number; GI, gastrointestinal.

**Table 2:** Risk of bias.

Author, Year	Bias Arising from the Randomization Process	Bias Due to Deviations from Intended Intervention	Bias Due to Missing Outcome Data	Bias in Measurement of the Outcome	Bias in Selection of the Reported Results	Overall Bias
Liu et al., 2021, China [10]	Some concern	Low risk	Low risk	Low risk	Low risk	Some concern
Tan et al., 2022, China [11]	Some concern	Low risk	Low risk	Low risk	Low risk	Some concern
Lu et al., 2022, China [13]	Low risk	Low risk	Low risk	Low risk	Low risk	Low risk
Hu et al., 2022, China [14]	Low risk	Low risk	Low risk	Low risk	Low risk	Low risk
Guo et al., 2022, China [12]	Low risk	Low risk	Low risk	Low risk	Low risk	Low risk
Ye et al., 2023, China [16]	Some concern	Low risk	Low risk	Low risk	Low risk	Some concern
Liu et al., 2023, China [15]	Some concern	Low risk	Low risk	Low risk	Low risk	Some concern

**Table 3:** Summary of the meta-analysis.

Outcome	No. of Studies	No. of Patients	Conventional Meta-Analysis		Trial Sequential Analysis			NNT
			RR or WMD, with 95% CI	Heterogeneity (I^2^/τ)	Conventional Test Boundary	Monitoring Boundary	RIS	
Sedation success	7	1502	Not significant (RR 0.999, 95% CI 0.995–1.003)	0.0/0.000	Not cross	Not cross	Exceed RIS (1502 of 599 patients) Enter futility border	Not significant (NNTH: 96; 95% CI NNTH 56 to ∞ NNTB 351)
Hypoxemia	6	1422	Significant (RR 0.302, 95% CI 0.187–0.489)	20.595/0.573	Cross	Cross	36.0% (1422 of 3951 patients)	Significant (NNTB: 13; 95% CI NNTB 10 to NNTB 20)
Hypotension	6	1422	Significant (RR 0.477, 95% CI 0.330–0.690)	72.634/0.593	Cross	Cross	53.6% (1422 of 2654 patients)	Significant (NNTB: 5; 95% CI NNTB 4 to NNTB 6)
Bradycardia	5	1323	Significant (RR 0.396, 95% CI 0.249–0.631)	47.484/0.722	Coss	Not cross	22.6% (1323 of 5851 patients)	Significant (NNTB: 16; 95% CI NNTB 11 to NNTB 29)
Respiratory depression	4	838	Significant (RR 0.347, 95% CI 0.183–0.659)	0.0/0.000	Cross	Not cross	26.9% (838 of 3118 patients)	Significant (NNTB: 17; 95% CI NNTB 11 to NNTB 36)
Time to LOC	4	952	Significant (WMD 5.385, 95% CI 2.082–8.689)	65.420/1.641	Cross	Cross	99.5% (952 of 957 patients)	NR
Recovery time	4	922	Not significant (WMD 0.573, 95% CI −0.705 to 1.851)	83.050/1.080	Not cross	Not cross	46.8% (922 of 1968 patients)	NR
Injection pain	4	838	Significant (RR 0.207, 95% CI 0.074–0.579)	2.823/0.628	Cross	Not cross	3.8% (838 of 22,319 patients)	Not significant (NNTB: 428; 95% CI NNTH 86 to ∞ NNTB 61)
Dizziness/headache	4	822	Not significant (RR 0.726, 95% CI 0.419–1.257)	0.0/0.000	Not cross	Not cross	17.2% (822 of 4774 patients)	Not significant (NNTB: 60; 95% CI NNTH 77 to ∞ NNTB 21)
PONV	4	1039	Not significant (RR 1.411, 95% CI 0.778–2.559)	0.0/0.000	Not cross	Not cross	10.4% (1039 of 9973 patients)	Not significant (NNTH: 74; 95% CI NNTH 28 to ∞ NNTB 111)

No., number; RR, relative risk; WMD, weighted mean difference; CI, confidence interval; LOC, loss of consciousness; PONV, postoperative nausea and vomiting; NNT, number needed to treat; RIS, required information size; NNTH, number needed to treat harm; NNTB, number needed to treat benefit; NR, not reported.

**Table 4:** GRADE evidence quality for each outcome.

Outcomes	Number of Studies	Quality Assessment					Quality
		ROB	Inconsistency	Indirectness	Imprecision	Publication Bias	
Sedation success rate	7	not serious	not serious	not serious	not serious	NA	⨁⨁⨁⨁ High
Hypoxemia	6	not serious	not serious	not serious	not serious	NA	⨁⨁⨁⨁ High
Hypotension	6	not serious	serious ^a^	not serious	not serious	NA	⨁⨁⨁𐤏 Moderate
Bradycardia	5	not serious	not serious	not serious	not serious	NA	⨁⨁⨁⨁ High
Respiratory depression	4	not serious	not serious	not serious	serious ^b^	NA	⨁⨁⨁𐤏 Moderate
Time to LOC	4	not serious	serious ^a^	not serious	serious ^b^	NA	⨁⨁𐤏𐤏 Low
Recovery time	4	not serious	serious ^a^	not serious	serious ^b^	NA	⨁⨁𐤏𐤏 Low
Injection pain	4	not serious	not serious	not serious	serious ^b^	NA	⨁⨁⨁𐤏 Moderate
Dizziness/headache	4	not serious	not serious	not serious	serious ^b^	NA	⨁⨁⨁𐤏 Moderate
PONV	4	not serious	not serious	not serious	serious ^b^	NA	⨁⨁⨁𐤏 Moderate

ROB, risk of bias; PONV, postoperative nausea and vomiting; NA, not applicable. GRADE Working Group grades of evidence. High certainty (⨁⨁⨁⨁): We are very confident that the true effect lies close to the estimated effect. Moderate certainty (⨁⨁⨁𐤏): We are moderately confident in the effect estimate; the true effect is likely to be close to the estimate of the effect, but there is a possibility that it is substantially different. Low certainty (⨁⨁𐤏𐤏): Our confidence in the effect estimate was limited; the true effect may be substantially different from the effect estimate. Explanations. ^a^ The 95% prediction interval is significantly wider than the 95% confidence interval. ^b^ The number of included studies and patients was small.

## Data Availability

Data is contained within the article and Supplementary Material.

## Supplementary Materials

The following supporting information can be downloaded at: https://www.mdpi.com/article/10.3390/jpm14030276/s1, Figure S1: Forest plot for studies comparing the effect of remimazolam to that of propofol on the sedation success rate; Figure S2: The trial sequential analysis for studies comparing the effect of remimazolam to that of propofol on the sedation success rate; Figure S3: Sensitivity analysis excluding one study at a time for the incidence of hypotension; Figure S4: Forest plot for studies comparing the effect of remimazolam to that of propofol on the incidence of bradycardia; Figure S5: The trial sequential analysis for studies comparing the effect of remimazolam to that of propofol on the incidence of bradycardia; Figure S6: Forest plot for studies comparing the effect of remimazolam to that of propofol on the incidence of respiratory depression; Figure S7: The trial sequential analysis for studies comparing the effect of remimazolam to that of propofol on the respiratory depression; Figure S8: Forest plot for studies comparing the effect of remimazolam to that of propofol on time to loss of consciousness; Figure S9: Sensitivity analysis excluding one study at a time for time to loss of consciousness; Figure S10: The trial sequential analysis for studies comparing the effect of remimazolam to that of propofol on the time to loss of consciousness; Figure S11: Sensitivity analysis excluding one study at a time for Recovery time; Figure S12: The trial sequential analysis for studies comparing the effect of remimazolam to that of propofol on the recovery time; Figure S13: Forest plot for studies comparing the effect of remimazolam to that of propofol on injection pain; Figure S14: The trial sequential analysis for studies comparing the effect of remimazolam to that of propofol on injection pain; Figure S15: Forest plot for studies comparing the effect of remimazolam to that of propofol on the incidence of dizziness/headache; Figure S16: The trial sequential analysis for studies comparing the effect of remimazolam to that of propofol on the incidence of dizzinewss/headache; Figure S17: Forest plot for studies comparing the effect of remimazolam to that of propofol on the incidence of PONV; Figure S18: The trial sequential analysis for studies comparing the effect of remimazolam to that of propofol on the incidence of PONV.
