# Supplementary material for: Remimazolam for Procedural Sedation in Older Patients: A Systematic Review and Meta-Analysis with Trial Sequential Analysis
Source: J Pers Med. 2024 Feb 29;14(3):276. doi: 10.3390/jpm14030276 (PMC10971009; doi:10.3390/jpm14030276)

Supplementary Figure S1. Forest plot for studies comparing the effect of remimazolam to that of propofol on the sedation success rate. The figure depicts individual trials as filled squares with relative sample size and the 95% confidence interval (CI) of the difference as a solid line. The diamond shape indicates the pooled estimate and uncertainty for the combined effect.

Study name	Statistics for each study		
	Risk ratio	Lower limit	Upper limit
Guo 2022	1.000	0.978	1.023
Hu 2022	0.994	0.978	1.010
Liu 2021	0.965	0.929	1.003
Liu 2023	0.973	0.939	1.008
Lu 2022	1.000	0.996	1.004
Tan 2022	1.002	0.981	1.023
Ye 2023	1.000	0.986	1.014
	0.999	0.995	1.003

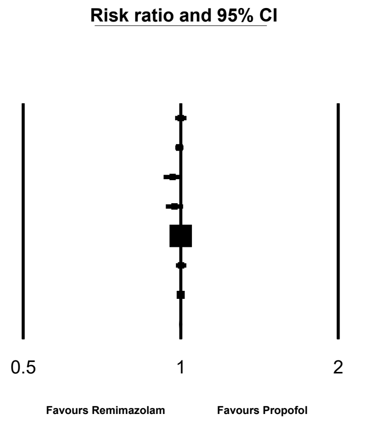

Supplementary Figure S2. **The trial sequential analysis for studies comparing the effect of remimazolam to that of propofol on the sedation success rate.** Uppermost and lowermost curves represent trial sequential monitoring boundary lines for benefit and harm respectively. Horizontal line represents the conventional boundaries for statistical significance. Triangular lines on the right side reflects the futility boundaries. The blue solid line represents the cumulative z-curve. The number on the x-axis indicates required information size.

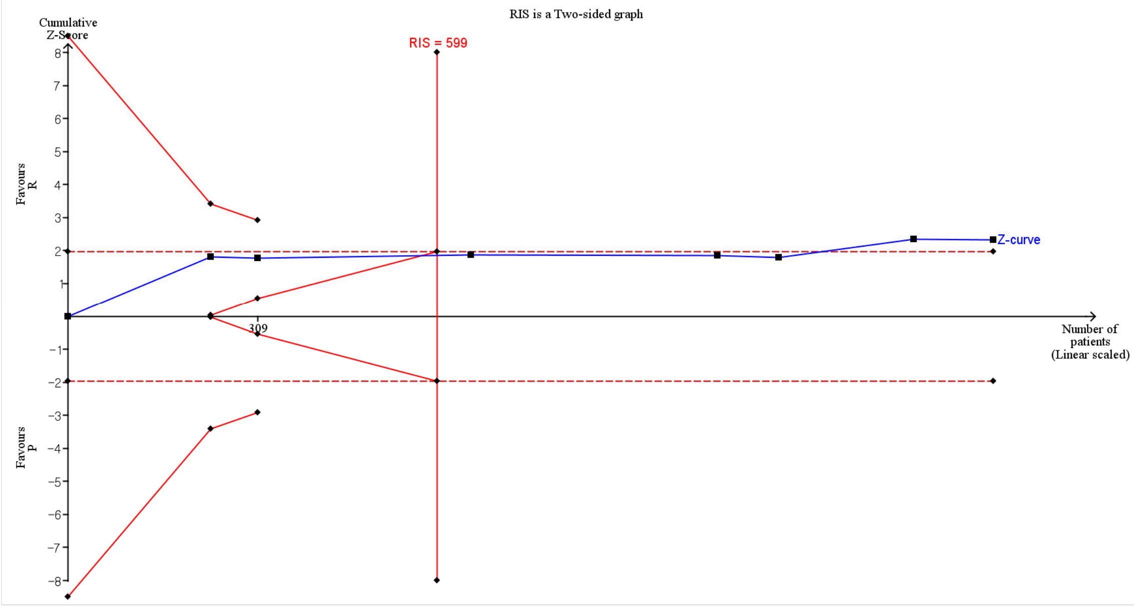

Supplementary Figure S3. Sensitivity analysis excluding one study at a time for the incidence of hypotension. The figure depicts individual trials as filled squares with relative sample size and the 95% confidence interval (CI) of the difference as a solid line. The diamond shape indicates the pooled estimate and uncertainty for the combined effect.

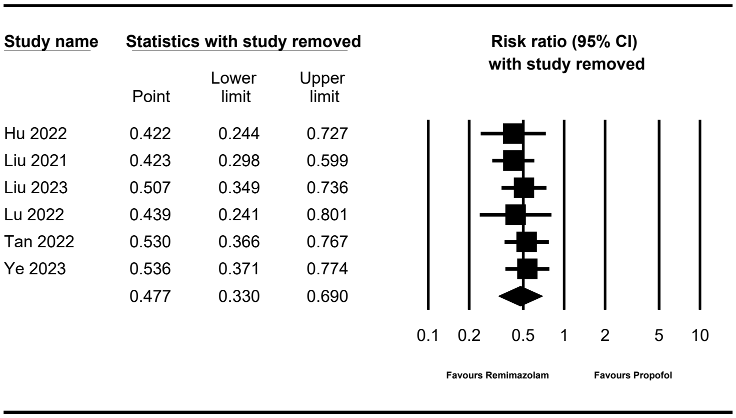

Supplementary Figure S4. Forest plot for studies comparing the effect of remimazolam to that of propofol on the incidence of bradycardia. The figure depicts individual trials as filled squares with relative sample size and the 95% confidence interval (CI) of the difference as a solid line. The diamond shape indicates the pooled estimate and uncertainty for the combined effect.

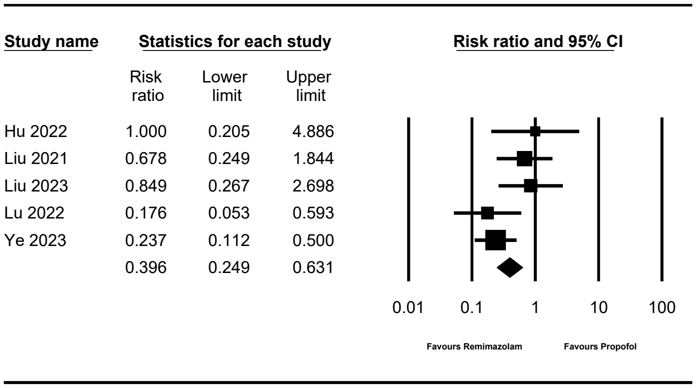

Supplementary Figure S5. The trial sequential analysis for studies comparing the effect of remimazolam to that of propofol on the incidence of bradycardia. Uppermost and lowermost curves represent trial sequential monitoring boundary lines for benefit and harm respectively. Horizontal line represents the conventional boundaries for statistical significance. Triangular lines on the right side reflects the futility boundaries. The blue solid line represents the cumulative z-curve. The number on the x-axis indicates required information size.

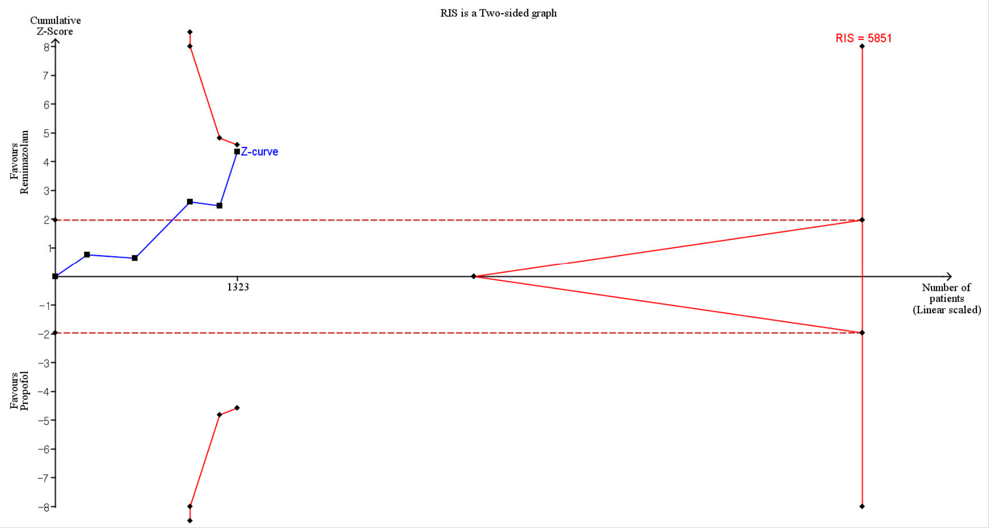

Supplementary Figure S6. Forest plot for studies comparing the effect of remimazolam to that of propofol on the incidence of respiratory depression. The figure depicts individual trials as filled squares with relative sample size and the 95% confidence interval (CI) of the difference as a solid line. The diamond shape indicates the pooled estimate and uncertainty for the combined effect.

Study name	Statistics for each study		
	Risk ratio	Lower limit	Upper limit
Guo 2022	0.217	0.050	0.938
Liu 2021	1.017	0.000	6494.351
Lu 2022	0.450	0.210	0.964
Ye 2023	0.127	0.016	0.986
	0.347	0.183	0.659

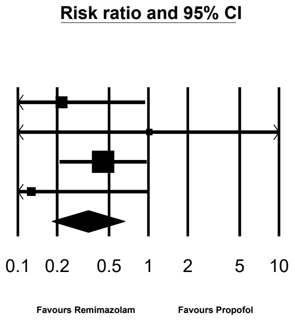

Supplementary Figure S7. The trial sequential analysis for studies comparing the effect of remimazolam to that of propofol on the respiratory depression. Uppermost and lowermost curves represent trial sequential monitoring boundary lines for benefit and harm respectively. Horizontal line represents the conventional boundaries for statistical significance. Triangular lines on the right side reflects the futility boundaries. The blue solid line represents the cumulative z-curve. The number on the x-axis indicates required information size.

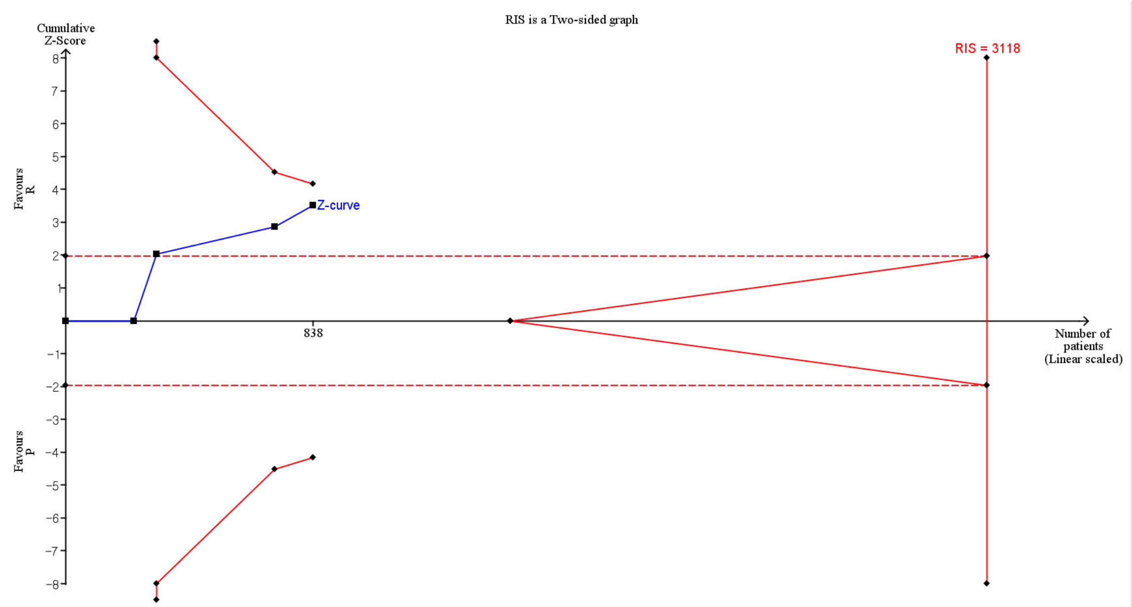

Supplementary Figure S8. Forest plot for studies comparing the effect of remimazolam to that of propofol on time to loss of consciousness. The figure depicts individual trials as filled squares with relative sample size and the 95% confidence interval (CI) of the difference as a solid line. The diamond shape indicates the pooled estimate and uncertainty for the combined effect.

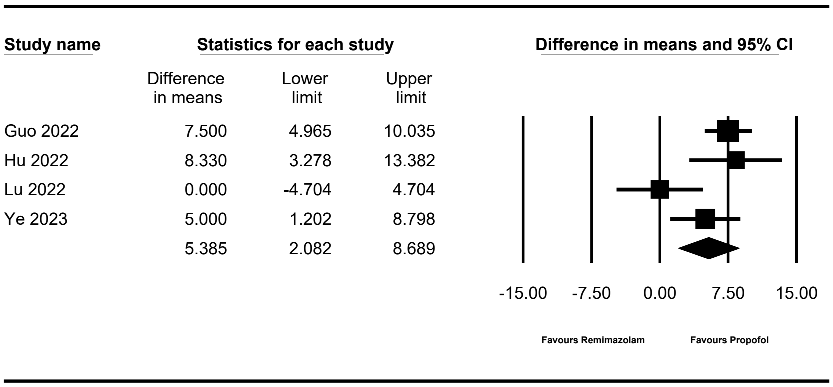

Supplementary Figure S9. Sensitivity analysis excluding one study at a time for time to loss of consciousness. The figure depicts individual trials as filled squares with relative sample size and the 95% confidence interval (CI) of the difference as a solid line. The diamond shape indicates the pooled estimate and uncertainty for the combined effect.

Study name	Point	Lower limit	Upper limit
Guo 2022	4.403	-0.011	8.817
Hu 2022	4.547	0.455	8.640
Lu 2022	6.967	5.021	8.913
Ye 2023	5.422	0.654	10.191
	5.385	2.082	8.689

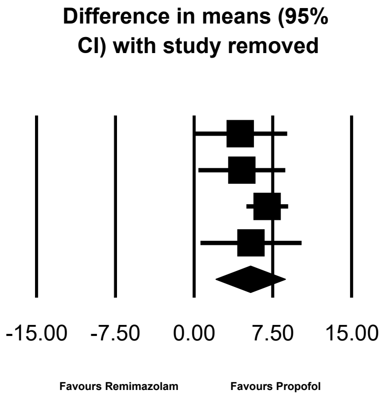

Supplementary Figure S10. The trial sequential analysis for studies comparing the effect of remimazolam to that of propofol on the time to loss of consciousness. Uppermost and lowermost curves represent trial sequential monitoring boundary lines for benefit and harm respectively. Horizontal line represents the conventional boundaries for statistical significance. Triangular lines on the right side reflects the futility boundaries. The blue solid line represents the cumulative z-curve. The number on the x-axis indicates required information size.

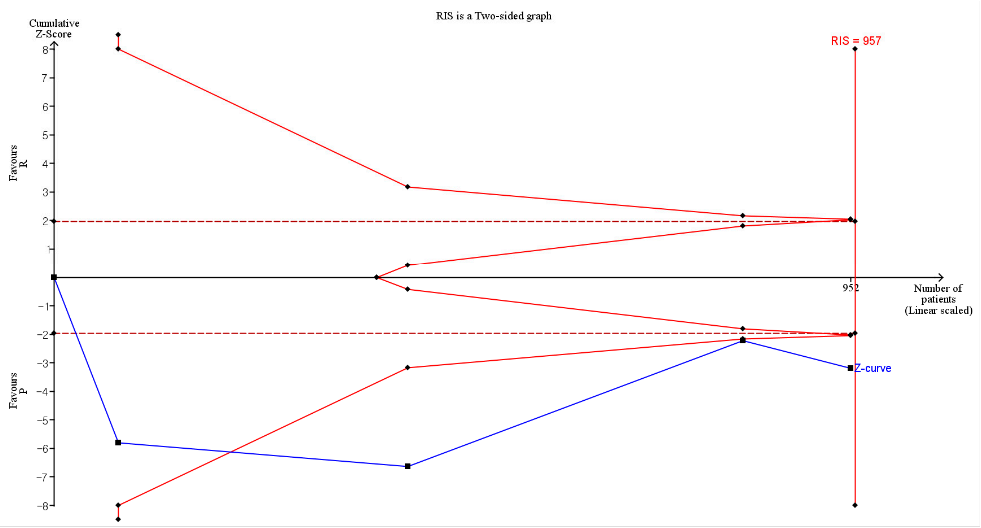

Supplementary Figure S11. **Sensitivity analysis excluding one study at a time for Recovery time.** The figure depicts individual trials as filled squares with relative sample size and the 95% confidence interval (CI) of the difference as a solid line. The diamond shape indicates the pooled estimate and uncertainty for the combined effect.

Study name	Point	Lower limit	Upper limit
Guo 2022	0.880	-0.638	2.398
Hu 2022	0.288	-1.363	1.939
Lu 2022	1.037	-0.202	2.275
Tan 2022	0.139	-1.227	1.505
	0.573	-0.705	1.851

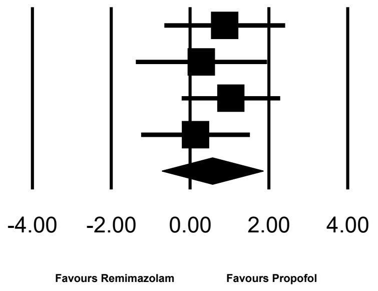

Supplementary Figure S12. The trial sequential analysis for studies comparing the effect of remimazolam to that of propofol on the recovery time. Uppermost and lowermost curves represent trial sequential monitoring boundary lines for benefit and harm respectively. Horizontal line represents the conventional boundaries for statistical significance. Triangular lines on the right side reflects the futility boundaries. The blue solid line represents the cumulative z-curve. The number on the x-axis indicates required information size.

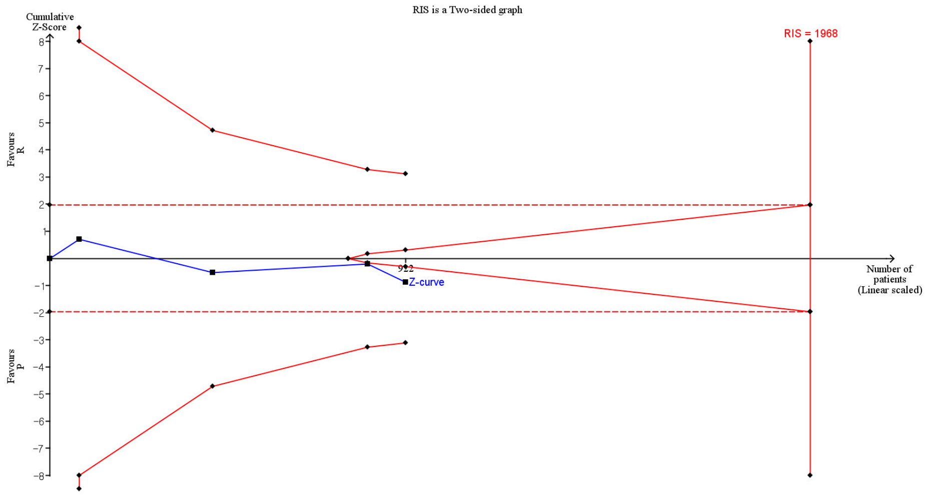

Supplementary Figure S13. Forest plot for studies comparing the effect of remimazolam to that of propofol on injection pain. The figure depicts individual trials as filled squares with relative sample size and the 95% confidence interval (CI) of the difference as a solid line. The diamond shape indicates the pooled estimate and uncertainty for the combined effect.

Study name	Statistics for each study		
	Risk ratio	Lower limit	Upper limit
Guo 2022	0.019	0.000	10.031
Liu 2021	0.271	0.093	0.793
Lu 2022	0.004	0.000	2.069
Ye 2023	0.013	0.000	6.427
	0.207	0.074	0.579

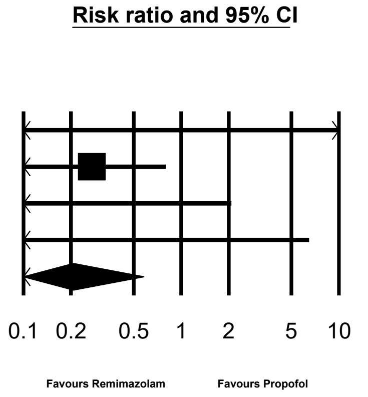

Supplementary Figure S14. The trial sequential analysis for studies comparing the effect of remimazolam to that of propofol on injection pain. Horizontal line represents the conventional boundaries for statistical significance. The blue solid line represents the cumulative z-curve. The number on the x-axis indicates required information size.

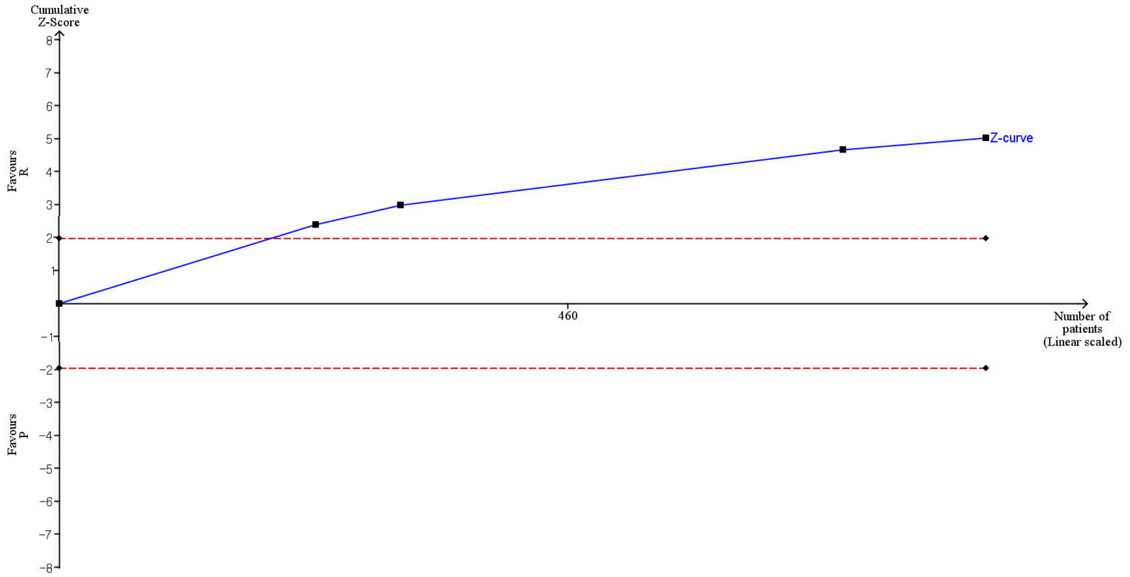

Supplementary Figure S15. Forest plot for studies comparing the effect of remimazolam to that of propofol on the incidence of dizziness/headache. The figure depicts individual trials as filled squares with relative sample size and the 95% confidence interval (CI) of the difference as a solid line. The diamond shape indicates the pooled estimate and uncertainty for the combined effect.

Study name	Statistics for each study			Risk ratio and 95% CI
	Risk ratio	Lower limit	Upper limit	
Liu 2023	1.019	0.261	3.969	
Lu 2022	1.000	0.000	6393.479	
Ye 2023	0.339	0.071	1.615	
Guo 2022	0.766	0.399	1.468	
	0.726	0.419	1.257	

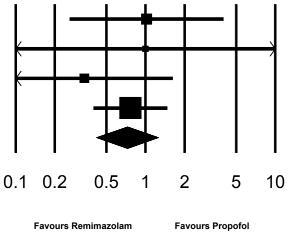

Supplementary Figure S16. The trial sequential analysis for studies comparing the effect of remimazolam to that of propofol on the incidence of dizzinewss/headache. Uppermost and lowermost curves represent trial sequential monitoring boundary lines for benefit and harm respectively. Horizontal line represents the conventional boundaries for statistical significance. Triangular lines on the right side reflects the futility boundaries. The blue solid line represents the cumulative z-curve. The number on the x-axis indicates required information size.

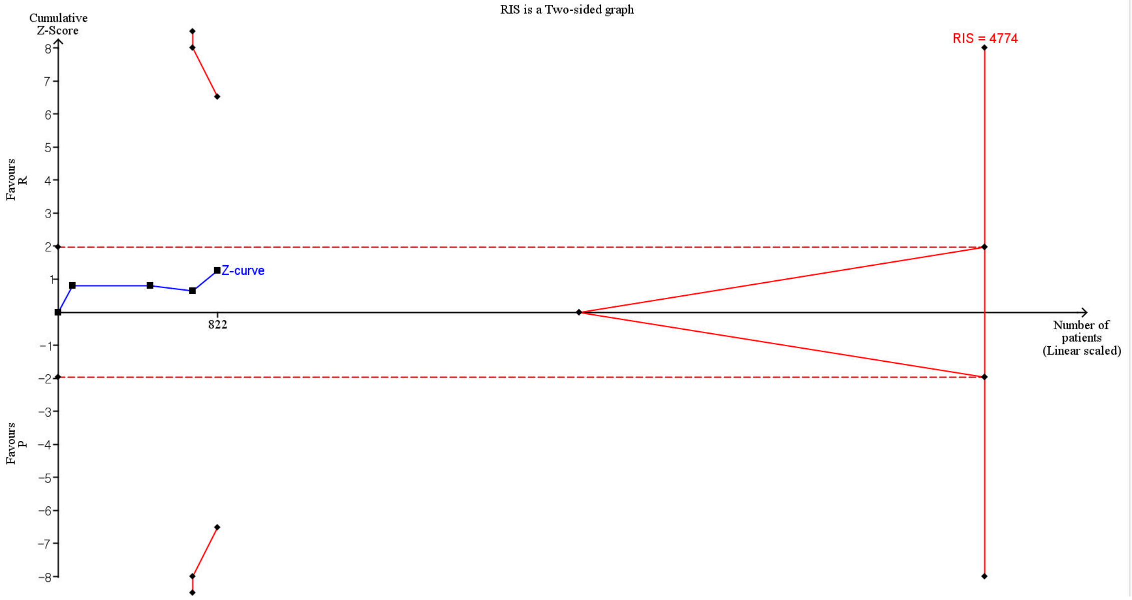

Supplementary Figure S17. Forest plot for studies comparing the effect of remimazolam to that of propofol on the incidence of PONV. The figure depicts individual trials as filled squares with relative sample size and the 95% confidence interval (CI) of the difference as a solid line. The diamond shape indicates the pooled estimate and uncertainty for the combined effect.

Study name	Statistics for each study			Risk ratio and 95% CI
	Risk ratio	Lower limit	Upper limit	
Guo 2022	1.218	0.539	2.752	
Hu 2022	3.000	0.123	73.136	
Liu 2023	1.601	0.645	3.974	
Lu 2022	1.000	0.000	6393.479	
	1.411	0.778	2.559	

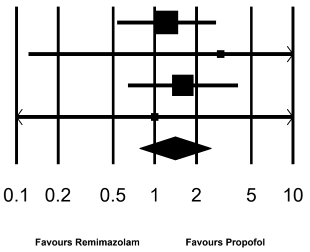

Supplementary Figure S18. The trial sequential analysis for studies comparing the effect of remimazolam to that of propofol on the incidence of PONV. Uppermost and lowermost curves represent trial sequential monitoring boundary lines for benefit and harm respectively. Horizontal line represents the conventional boundaries for statistical significance. Triangular lines on the right side reflects the futility boundaries. The blue solid line represents the cumulative z-curve. The number on the x-axis indicates required information size.

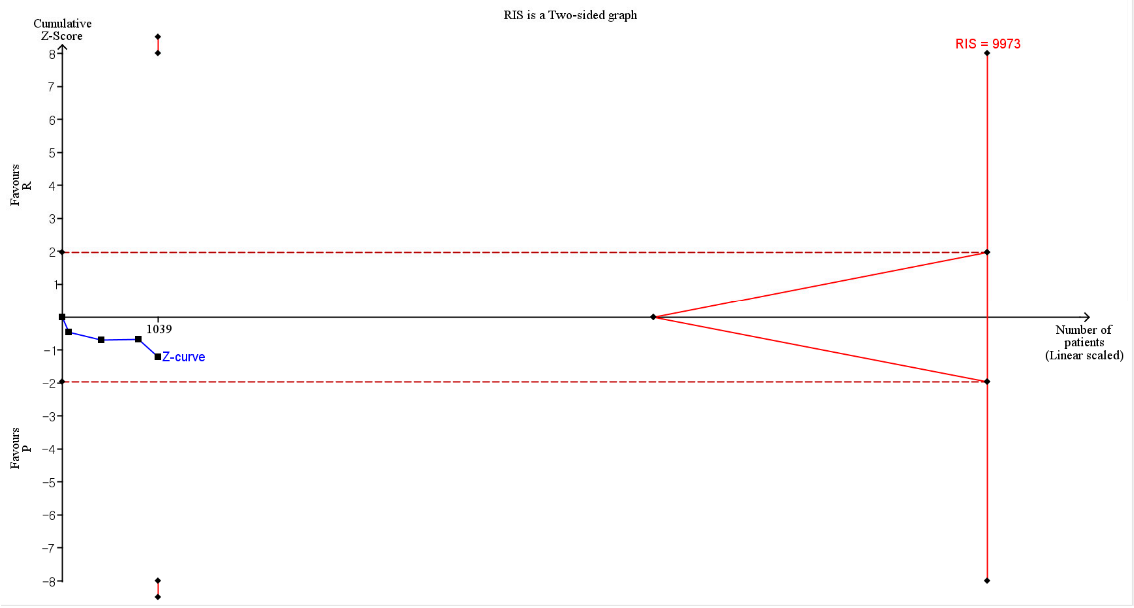

Supplement: Supplementary file 1 [file jpm-14-00276-s001.zip › Supplementary Figures.pdf]
